# Reconstruction of massive tibial bone and soft tissue defects by trifocal bone transport combined with soft tissue distraction: experience from 31 cases

**DOI:** 10.1186/s12891-020-03894-y

**Published:** 2021-01-07

**Authors:** Yong-Qing Xu, Xin-Yu Fan, Xiao-Qing He, Hong-Jie Wen

**Affiliations:** Department of Orthopaedic Surgery, 920th Hospital of Joint Logistics Support Force, Kunming Medical University, 212 Daguan Road, Xi Shan district, Kunming, Yunnan People’s Republic of China 650031

**Keywords:** Ilizarov techniques, Bone transport, Soft-tissue transport, Tibia, Defects

## Abstract

**Background:**

Large post-traumatic tibial bone defects combined with soft tissue defects are a common orthopedic clinical problem associated with poor outcomes when treated using traditional surgical methods. The study was designed to investigate the safety and efficacy of trifocal bone transport (TFT) and soft-tissue transport with the Ilizarov technique for large posttraumatic tibial bone and soft tissue defects.

**Methods:**

We retrospectively reviewed 31 patients with massive posttraumatic tibial bone and soft tissue defects from May 2009 to May 2016. All of the eligible patients were managed by TFT and soft-tissue transport. The median age was 33.4 years (range, 2–58 years). The mean defect of bone was 11.87 cm ± 2.78 cm (range, 8.2–18.2 cm) after radical resection performed by TFT. The soft tissue defects ranged from 7 cm × 8 cm to 24 cm × 12 cm. The observed results included bone union time, wound close time and true complications. The Association for the Study and Application of the Method of Ilizarov (ASAMI) scoring system was used to assess bone and functional results and postoperative complications were evaluated by Paley classification.

**Results:**

The mean duration of follow-up after frame removal was 32 months (range, 12–96 months). All cases achieved complete union in both the elongation sites and the docking sites, and eradication of infection. The mean bone transport time was 94.04 ± 23.33 days (range, 63.7–147 days). The mean external fixation time was 22.74 ± 6.82 months (range, 14–37 months), and the mean external fixation index (EFI) was 1.91 ± 0.3 months/cm (range, 1.2–2.5 months/cm). The bone results were excellent in 6 patients, good in 14 patients, fair in 8 patients and poor in 3 patients. The functional results were excellent in 8 patients, good in 15 patients, fair in 5 patients and poor in 3 patients. Conclusion: TFT, in conjunction with soft tissue transport technique, can give good results in most patients (in this article, good and excellent results were observed in 64% of patients). Soft tissue transport is a feasible method in providing good soft tissue coverage on the bone ends. Although it has no advantages over microvascular techniques, it might be an good alternative in the absence of an experienced flap surgeon. Nonetheless, high-quality controlled studies are needed to assess its long-term safety and efficacy.

## Background

Large posttraumatic bone defects of the tibia combined with soft tissue defects are a common clinical problem. Currently, the treatment methods mainly include bone transport, the Masquelet technique, and free vascularised bone transfer [1–9]. For decades, bone transport has become recognised as an effective method for the management of large posttraumatic tibial and soft tissue defects because of the complete eradication of infection, and powerful capacity for osteogenesis.

However, bifocal bone transport (BFT), which is regarded as a simple-level osteotomy bone transport, has the disadvantages of long frame duration, poor regenerates, and frequent complications. In recent decades, some scholars proposed the concept of TFT, which is also called double level osteotomy bone transport, and is strictly only suitable for bone defects over or equal to 8 cm [10, 11]. At present, there are few clinical reports about this technique. Its indications, advantages over single level osteotomy, and long-term complications are still unclear. Previously, the studies of applying distraction to simultaneously manage posttraumatic long tibial defects composited with massive soft tissue defects are really rare. In addition, the argument of whether it is important to restore soft-tissue envelop before bone transport is always there.

In this series, we assess the results of TFT in concert with soft-tissue transport in management of posttraumatic large tibial bone loss composited with soft tissue defects.

## Methods

### Inclusion and exclusion criteria

Inclusion: i) The bone defect of the tibia caused by trauma was ≥ 8 cm after debridement, and combined with large soft tissue defects (> 15cm^2^); ii) All of the tibial bone, including bilateral ends of the bone defects and osteotomy sites, were covered with soft tissue after debridement; iii) The soft tissue wound, which has no exposed bone, was managed by soft-tissue transport; iv) Patients were aged 18–65;v) Follow-up was longer than 2 years.

Exclusion: i) Bilateral tibial bone defect; ii) Soft tissue wound was managed by flap graft; iii) diabetic/corticosteriod treated patients, who are susceptible to infection and non-union.

### Demographic data

A total of 31 cases were eligible, including 27 males and 4 females, with a mean age of 33.4 years (range, 18 to 58). There were 20 affected limbs on the right and 11 on the left. Fifteen cases were injured by traffic accident, 12 were machine injuries and 4 were crushed by stones. The fracture classification of all the patients was identified as Gustilo IIIB. The mean length of tibia defects was 11.4 cm (range, 8 to 18.2). The mean soft tissue defect was 42.72 cm^2^ (ranged, 56–288 cm^2^). Radiographs, blood test and bacterial culture were performed on each patient. More than three independent samples were obtained from the wound after osteotomy and debridement and used for bacterial culture. The following blood tests were performed: white blood cell level, C-reactive protein, and erythrocyte sedimentation rate, and these data are reported in Table 1.

**Table 1:**
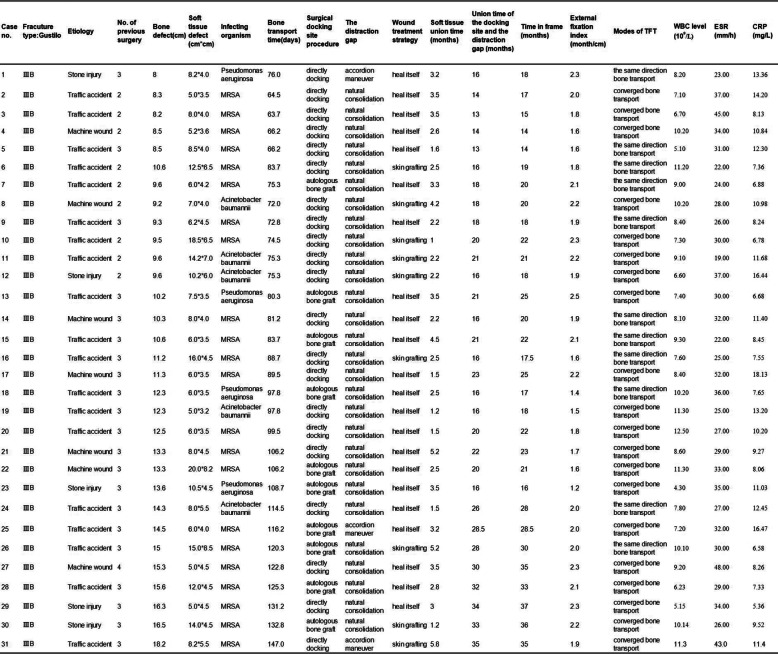
Demographic and baseline data of the study patients

### Surgical technique

**Fig. 1:**
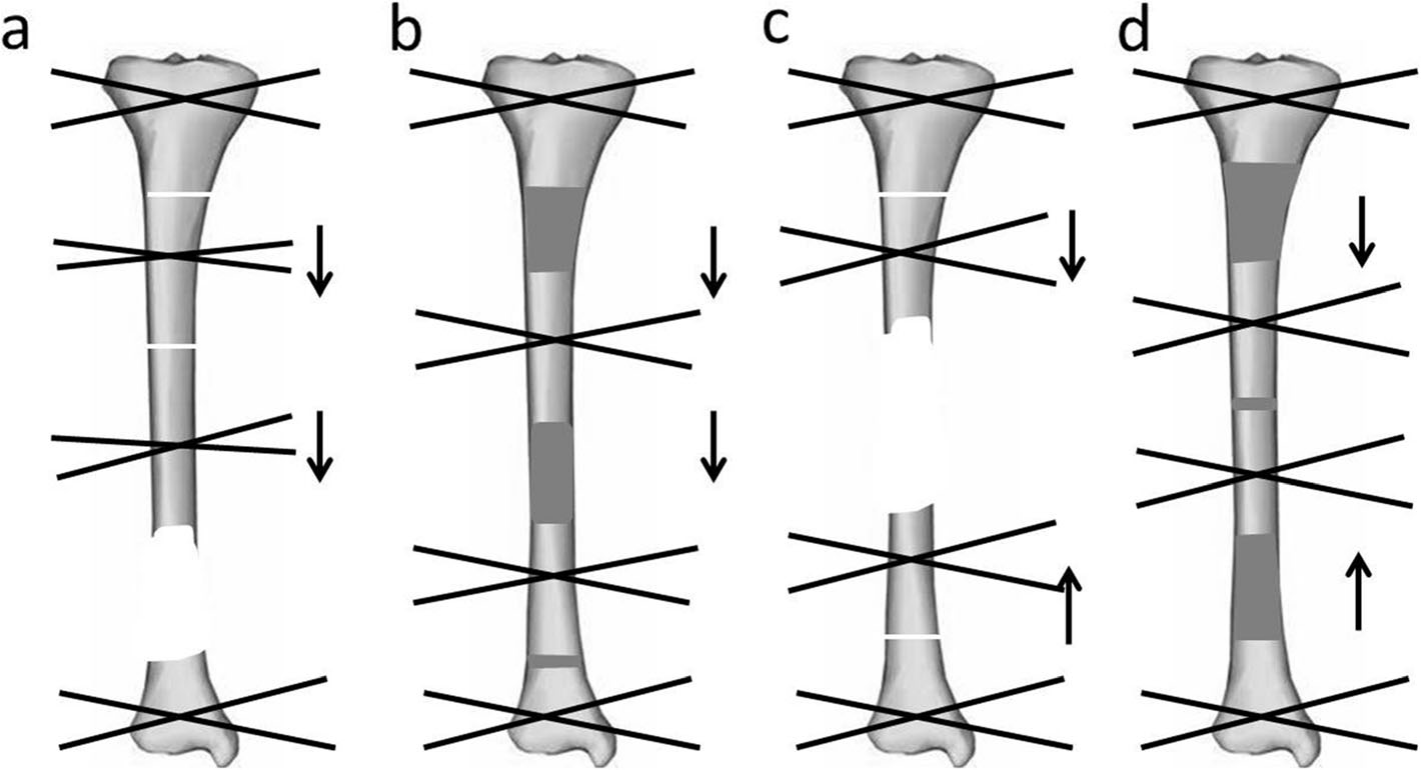
Illustration showing the modes of trifocal bone transport: **a**-**b** the same direction bone transport **c**-**d** the converged bone transport

**Fig. 2:**
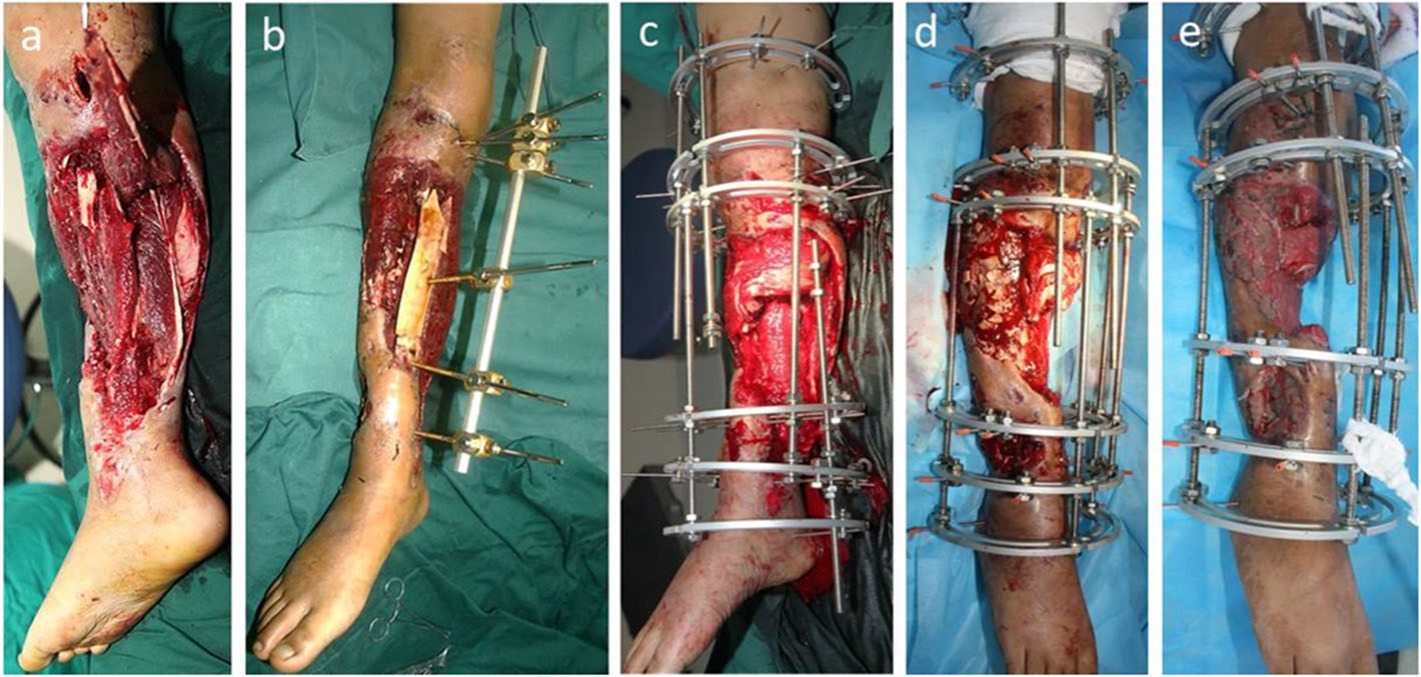
**a** Appearance of the injured right lower leg, open fracture and Gustilo IIIB. **b** After debridement and external fixation in local hospital, the patient was transferred to our center, with long bone exposure and large soft tissue defect. **c**-**d** 15 days after the injury, the patient underwent the first debridement in our hospital, and the previous external fixator was replaced with Ilizarov fixation. The necrotic soft tissue and bone was thoroughly debrided, and skin grafts were performed on partial wound. **e** Two weeks after the operation, skin graft survived, and most of the soft tissue wound was closed

**Fig. 3:**
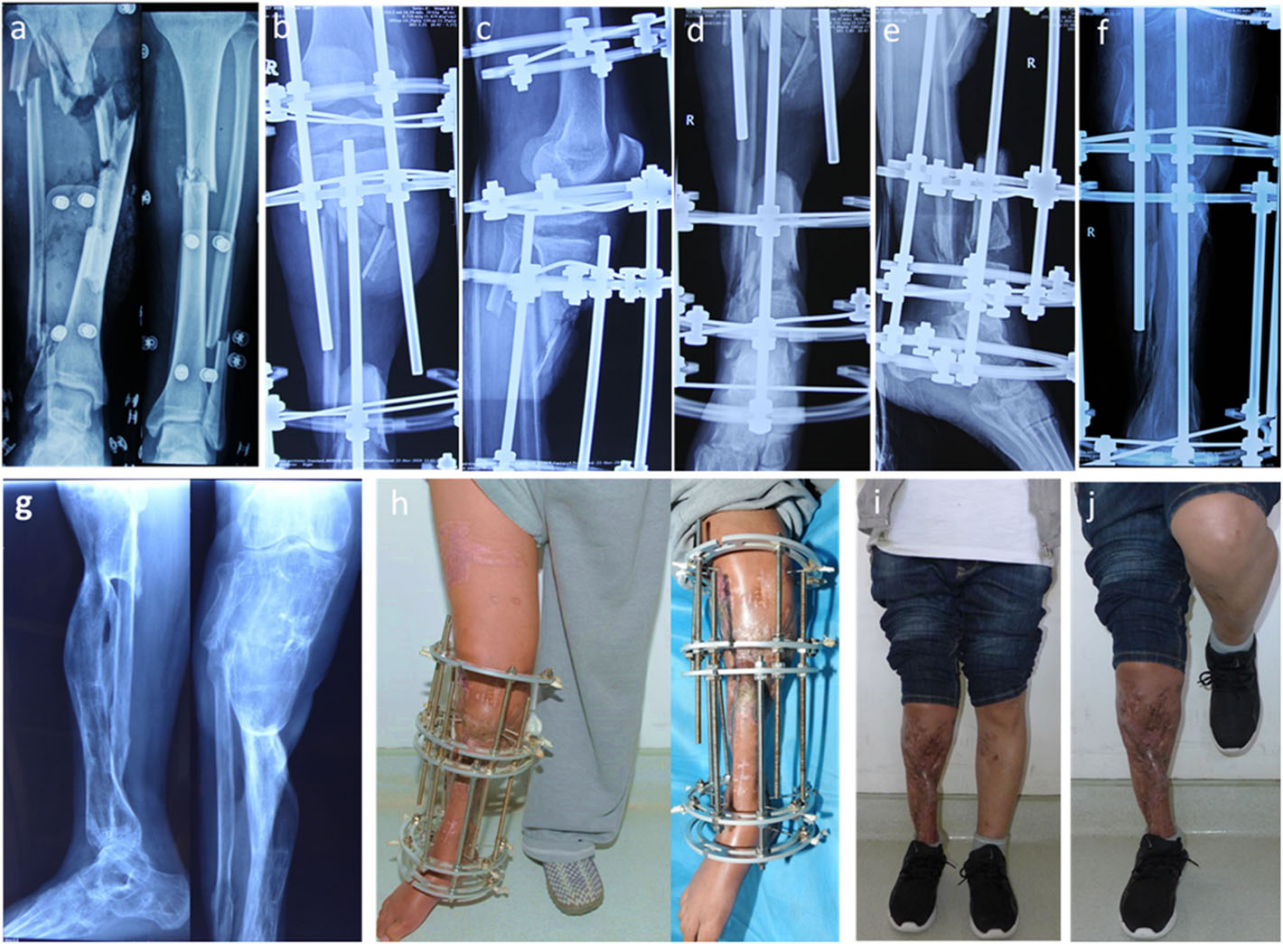
**a** Radiograph of case no. 31 who was involved in a traffic accident resulting in right tibia open fracture and Gustilo IIIB. **b**-**e** After three previous debridement operations, there was a defect of 18.2 cm on the tibia, and the proximal and distal osteotomy was performed. The osteotomy sites were obviously shown on the X-rays. **f** Radiograph of transport bone segment reaching docking site 30 months after osteotomy. **g** Union was achieved both in docking site and elongation area 35 months post-operatively. **h** Soft tissue defect was closed during the process of bone transport 5.8 months after osteotomy. **i**-**j** Complete bone and soft tissue union was achieved and frame was removed 35 months after osteotomy

**Fig. 4:**
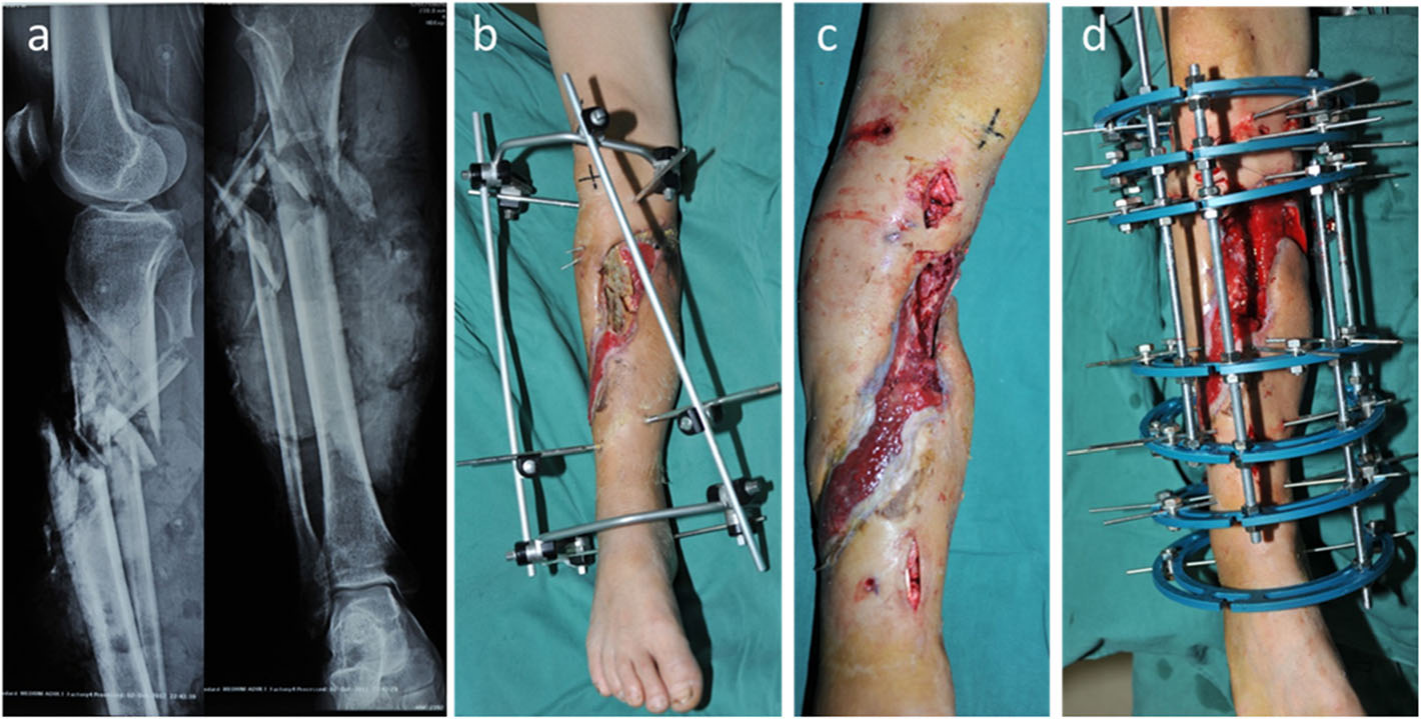
**a** Radiograph of case no. 27 who was involved in a traffic accident resulting in right tibia open fracture and Gustilo IIIB. **b** Three weeks later, the patient was transferred to our center and there was lager soft tissue defects associated with facture. **c**-**d** The patient underwent the first debridement in our hospital 23 days after injury, and the previous external fixator was replaced with Ilizarov fixation. The wound was open after the necrotic soft tissue and bone was completely debrided, and treated with soft tissue distraction

**Fig. 5:**
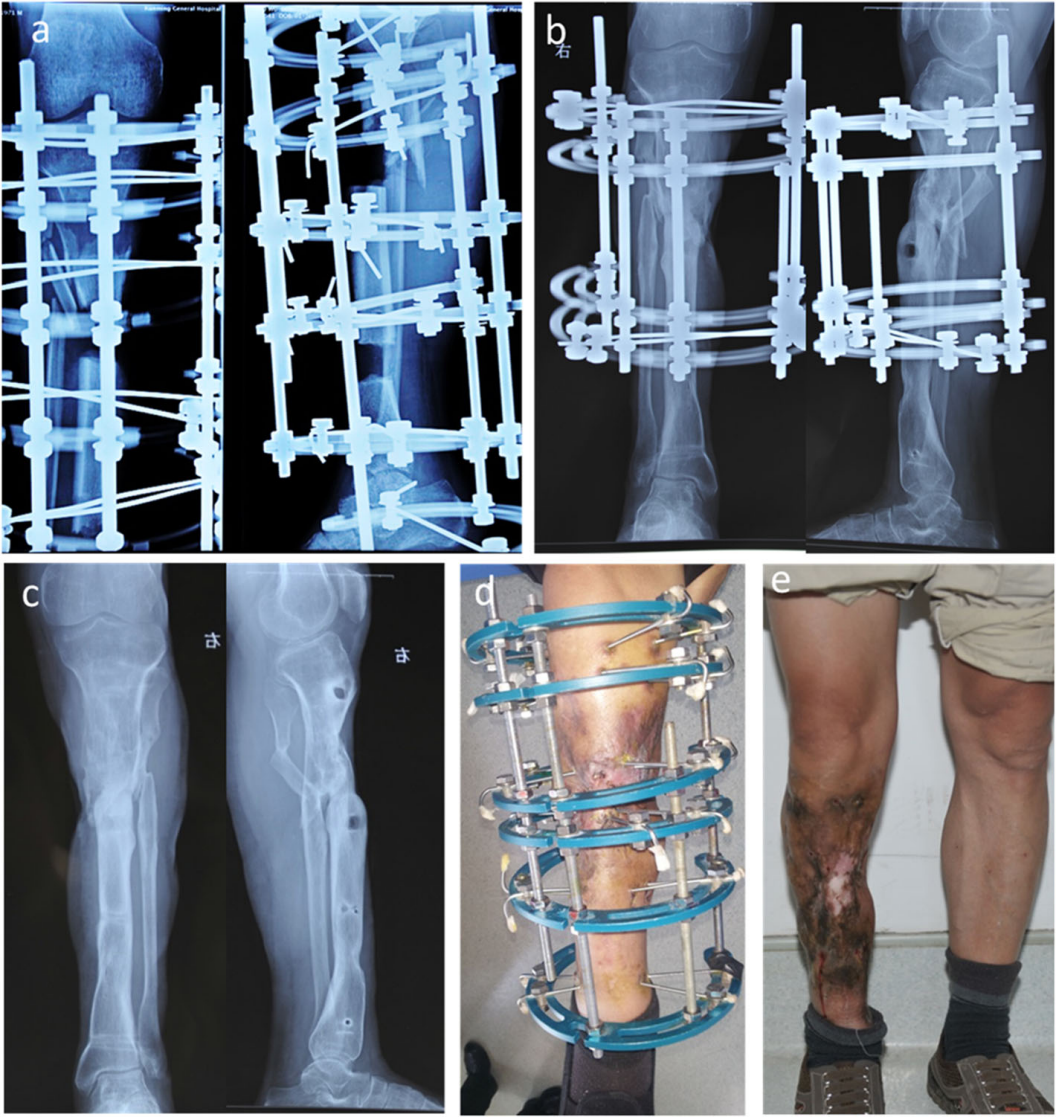
**a** Radiographs of distal and proximal osteotomy after meticulous debridement. The converged bone transport was performed on first day postoperative, and the proximal bone segment was transported at the rate of 1 mm/d, while the distal segment was transported at the speed of 0.6 mm/d, which was manipulated once in the morning and once in the evening. Two weeks later, the transport speed slowed down to 0.5 mm/d to 0.6 mm/d. **b** The docking site union and consolidation of regenerates were achieved 32 months after the operation. **c** Frame was removed with good bone result on 33 months after osteotomy. **d**-**e** Photographs of standing position with excellent functional result

The patient was placed in the supine position. Under general or regional anesthesia, the operation was performed based on the principle of “the clean area first, then the polluted area” [12] to avoid cross-infection. Firstly, two osteotomies were undertaken with electric saw and osteotome on the proximal and distal tibia. In this step, we performed a 1.5 cm to 2 cm incision and carefully protected the skin, subcutaneous tissues and periosteum. Then, the osteotomy sites were wrapped with iodine gauze. Usually, the Ilizarov fixation was installed in the previous operation. All internal fixation, necrotic bone, and infectious soft tissue were completely removed. Furthermore, meticulous debridement was performed until the appearance of fresh blood on the surfaces of the bone and soft tissue [13]. Repeated irrigation was performed using hydrogen peroxide solution, 0.9% normal saline, and iodophor. Both bone ends were trimmed with an electric saw to obtain smooth and sufficient healthy soft tissue coverage. Restoration of limb length and axis was achieved when installing a half-ring external fixator or the Ilizarov external fixator. The osteotomy site gap was immediately extended 0.3–0.5 cm, which would shorten the duration of distraction but not affect osteogenesis. Finally, the soft tissue wound was confirmed to be open and wrapped with iodine gauze. The soft tissue wound was repaired using soft tissue transport-a process wherein, along with bone distraction, the skin and subcutaneous tissue are stretched using the pins and screws of the Ilizarov ring. The soft tissue defect was then covered with new skin tissue before the bone ends contact at the docking site. Furthermore, in our series, TFT was performed without involving the fibula. The modes of TFT are illustrated in the diagrams (Fig. 1). In addition, the manipulation and process are illustrated in more detail in Figs. 2, 3, 4 and 5.

### Postoperative management

It was necessary to keep the pin-tract and surrounding skin clean and sterilise them with 75% ethanol, usually once a day. Frequent assessments of blood circulation, sensation and movement of the affected limbs and toes were also important. In the series, we filled the wound with iodophor gauze and changed the dressing regularly. On the first postoperative day, if no vascular or nerve crisis appeared at the distal of the affected limbs, the proximal bone segment was transported at the rate of 1 mm/d, while the distal segment was transported at the speed of 0.6 mm/d, by operating once in the morning and once in the evening. Notably, the transport speed was reduced to 0.5–0.6 mm/d after 2 weeks to relieve the patient’s pain. Six weeks later, the speed was adjusted according to the osteogenesis condition. During the process of distraction, if patients felt intense pain, the distraction was stopped for 1 week. When bone ends reaches docking sites, autologous bone grafts were performed if the ends were sharp or contacted narrowly. However, if the bone ends contacted widely, the protocol of dynamising the frames for 3 months is performed firstly. If no obvious callus was then seen at the docking site, an autologous bone graft was performed immediately. The radiological criterion of bone union was bridging of the docking site at three cortices observed on the anteroposterior and lateral radiographs. The clinical criterion of bone union was the ability to weight-bear fully without pain. The bone and functional results were assessed according to ASAMI classification [14] and complications were classified according to Paley classification [15].

## Results

### General results

The details of all patients are shown in Table 1.The mean follow-up was 32 months (range, 12 to 96 months). All cases achieved complete union both in soft tissue and bone defects. The limb length of 28 patients was completely restored, while a discrepancy of 1 cm to 2 cm was observed in 3 patients. The mean healing time of soft tissue wounds was 2.86 ± 1.22 months (range, 1.0–5.8 months). The mean bone union time was 20.98 ± 6.59 months (range, 14 to 35). Six patients were identified with docking site delayed union and four cases with docking site nonunion. We treated four delayed union patients with bone ends trimming without bone graft and six delayed union or nonunion patients with autologous bone graft. The mean bone transport time was 94.04 ± 23.33 days (range, 63.7–147 days). The mean soft tissue defects was 42.72 cm^2^ (ranged, 56–288 cm^2^). Nine patients were addressed with skin grafting and 22 patients healed by soft-tissue transport. The mean external fixation time was 22.74 ± 6.82 months (range, 14–37 months), and the mean external fixation index was 1.91 ± 0.3 months/cm (range, 1.2–2.5 months/cm).

### ASAMI score

The bone and functional results were assessed according to ASAMI classification at last visit (mean of 32 months) and was summarized in Table 2. The bone results were excellent in 6 patients, good in 14 patients, fair in 8 patients and poor in 3 patients. The functional results were excellent in 8 patients, good in 15 patients, fair in 5 patients and poor in 3 patients.

**Table 2:**
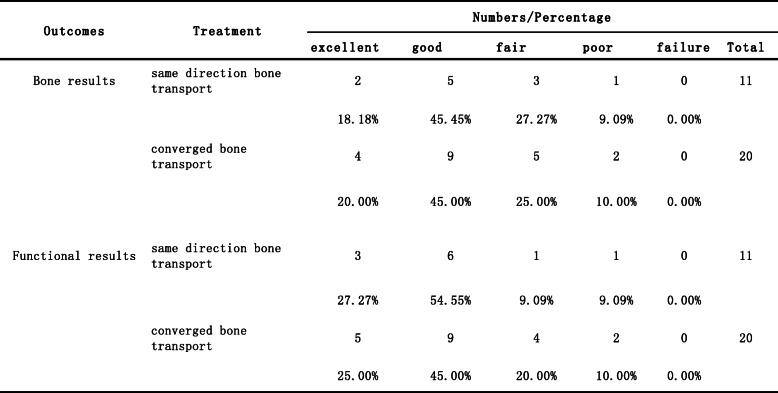
Evaluation of the bone and functional results according ASAMI classification

### Complications

Complications were classified according to Paley classification and detailed data were reported in Table 3. Muscle contraction was encountered in nine cases and resolved by physiotherapy, or Achilles tendon lengthening or applying apparatus. Three patients showed infection and poor osteogenesis in the distraction area, and this was treated with a vancomycin cement rod for 2 months and accordion maneuver. Four patients showed axial deviation, which disappeared after adjusting the frame. Two patients suffered severe pin-tract infection, which was addressed by dressing changes and oral antibiotic treatment. Six patients were identified with docking site delayed union and four cases with docking site nonunion. We treated four delayed union patients with bone ends trimming without bone graft and six delayed union or nonunion patients with autologous bone graft. One case suffered re-fracture on the docking site after removal of the frame and was treated by plate internal fixation and autologous bone graft. One showed K-wire cut out and healed after medical treatment and weight bearing. Joint stiffness either in knee or ankle occurred in 12 cases (38.70%) in our study and most of the cases was successfully improved by positive physiotherapy or extending apparatus. The details of complications are shown in Table 3.

**Table 3:**
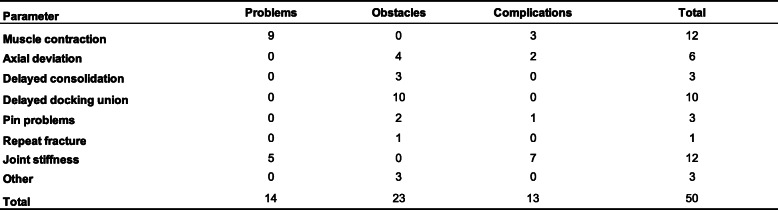
Complications in 31 trifocal tibial bone transport by Paley criteria

## Discussion

### Treatment for tibia bone and soft tissue defects

Options for tibia bone and soft tissue defects are varied. At present, the acute shortening and re-lengthening technique (AST) and bone transport are two common methods to treat posttraumatic tibial bone and soft tissue defects using an external fixator. AST is a satisfactory management method for tibia defects less than or equal to 5 cm, and has the advantage of a shortened healing time and easy control of axial deviation. However, vascular or nerve compromise frequently occurs when AST are performed on the patients of tibia defects > 5 cm [1, 3, 16]. Fortunately, bone transport can avoid limb discrepancy, contracture, blood circulation obstacles, and soft tissue incarceration, which are often encountered with the AST technique.

Currently, large post-traumatic tibial bone defects can be managed with BFT or FTT. According to previous research, when treating bone defects sized > 6 cm, the TFT technique may shorten the time of distraction, shorten the healing time of soft tissue defect, and reduce the rate of complications [17–19]. Paley et al. stated that TFT achieved better results in cases of bone defect > 10 cm as compared to the other Ilizarov technique [20]. Chevardin et al. [21] showed that the risk of hypoplastic bone formation increased in case of BFT regeneration of > 5 cm, and delayed osteogenesis occurred when the regeneration reached 8–10 cm. In our series, the mean bone defect size was 11.4 cm (range, 8–18.2). In our experience, for tibia defects ranging from 6 to 8 cm, the BFT technique is the most preferred approach because of the relatively simple manipulation. However, for tibia defects sized > 8 cm, the TFT technique is optimal.

### Bone transport combined with soft-tissue transport (open bone transport)

**Fig. 6:**
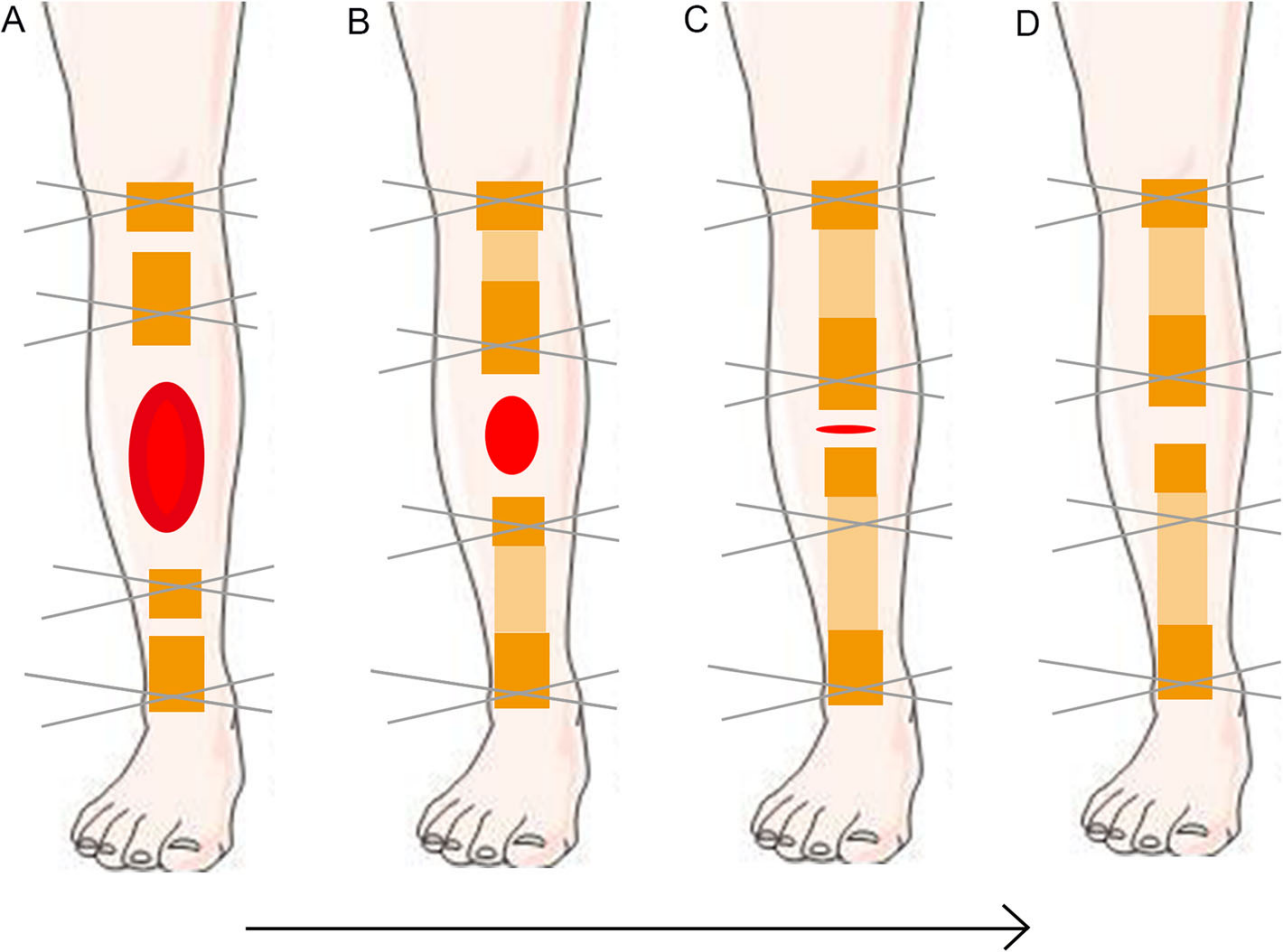
Illustration showing the healing process of wound by open bone transport technique. This picture was provided by Hongjie Wen, who was the corresponding author

Previously, the Ilizarov external fixation technique was the most commonly used technique for large bone defects without soft tissue defects [8, 22]. In recent years, an increasing number of studies have reported that bone transport with external fixation can be used to simultaneously treat massive bone and soft tissue defects [5, 9, 23, 24]. The technique of bone transport combined with soft-tissue transport (open bone transport) was first proposed by Suger [25]. It is a process of the skin and subcutaneous tissue be stretched by pins and screws of Ilizarov ring along with the bone distraction. Then, the soft-tissue defect was covered by the new formed skin tissue before the bone ends contact at the docking site. In 2000, Paley et al. [21] reported a retrospective trial of tibial bone defect treatment. Seven of the eight soft tissue defects were closed with soft-tissue transport and achieved healing. Then, several other scholars also reported this technique when treating tibial bone loss and soft-tissue defect [26–28]. This technique emphasises no flap transfer to cover the wound when performing bone transport to manage bone and soft-tissue defects which has no bone exposure after debridement. Regular dressing changes are needed for the wound, usually once a day (Fig. 6). The technique offers the advantage of simultaneously reconstructing both bone and soft-tissue defects with distraction, avoiding the procedure of flap. However, this technique also has the disadvantages of a long duration of regenerate consolidation and frame wearing, regenerated scarred soft tissues and frequent dressing change [29]. Paley et al. [21] stated that bone transport beneath the flap seemed to proceed more easily than in closed defects with scarred soft tissues and flap coverage may contribute to docking site union without grafting. The mean EFI in this series was 1.91 ± 0.3 months/cm, inferior to that reported by recent research studies on bone transport and flap technique [10, 11]. In addition, this technique is only suitable for the cases with both ends with good soft-tissue coverage and without bone exposure after debridement. In other cases, the bone ends will protrude through the wound during bone transport [24]. Finally, this technique is a feasible method to simultaneously manage large bone and soft-tissue defects without flap graft; however, its safety and efficacy warrant further investigation.

### Complications

Non-union, delayed union, re-fracture, recurrence of infection, infection of new forming bone, poor osteogenesis and axis deviation are common complications of bone transport [3, 5, 9, 10, 30]. Flat and wide bone ends may contribute to the stability of the docking site because of decreasing shear force. Therefore, it is easy to obtain union at the docking site without bone grafting. If the bone ends are sharp or contact narrowly, bone grafting may be required; otherwise, bone grafting should be performed only if there is no obvious callus formation after 3 months. In this study, delayed union and nonunion were defined as no obvious callus observed at the docking site at 3 and 6 months, respectively, after bone ends contact at the docking site. Six patients were identified with docking site delayed union and four cases with docking site nonunion. We treated four delayed union patients with bone ends trimming without bone graft and six delayed union or nonunion patients with autologous bone graft. Moreover, poor osteogenesis in the elongation area was observed in three cases. They were treated using the accordion maneuver [29] that involves repeatedly compressing and distracting the lengthening segments or were managed using autologous iliac cancellous bone. Notably, bone end trimming with an electric saw can cause mechanical and thermal damage to the bones, resulting in prolonged bone healing and complications. Therefore, osteotome could be a better alternative to an electric saw. Open bone grafting, which opens the soft-tissue defects, accompanied with an external fixator and vacuum-assisted wound closure (VAC), is also a feasible method that facilitates rich vascularization so encourages fast healing. If there is skin embedded between bone ends, relaxation surgery should be performed immediately [30, 31].

Previously, few studies have reported the technique of bone transport combined with soft-tissue transport because of the concern of increased risk of infection. In the series, 3 out of 31 (9.7%) subjects developed deep infection because the osteotomy was too close to the wound. Thus, the performance of osteotomy at a site that is far away from the wound increases the chances of success. In addition, the nursing quality of the osteotomy incision, such as dressing changes every 2 days and keeping it dry, plays a crucial role in avoiding contamination of the osteotomy sites. The presence of pus in the infectious bone area should be an indicator of delayed osteotomy in the metaphyseal. The two-step protocol implied in our series could significantly reduce the infection rate. First, complete debridement and VAC drainage were undertaken. Second, osteotomy and distraction were performed after fresh granulation tissue formation. Moreover, according our centre and other authors’ experience, placing vancomycin cement rods on the infectious site for one to 2 months can effectively control the infection [32, 33].

A total of four cases had the problem of axial deviation and the main reason was that the axial line is poor when placing the frame and the patients were not followed up in time. The prevention of axial deviation requires experienced surgeon to perform limb axial alignment under C-arm. Moreover, patients should be closely followed-up after the operation so that problems can be addressed in a timely fashion.

### Time in frame

Owing to the prolonged time in frame, most patients always complain when large post-traumatic bone defects are treated using the bone transport technique. In the study, the mean external fixation time was 22.74 ± 6.82 months (range, 14–37 months). More and more studies report bone transport over an intramedullary nail for reconstruction of long bone defects in the tibia, which can reduce the total time in frame [5, 34–41]. Lin et al. [41] reported a study of infectious tibial bone defects, and osteotomy and bone transport were performed after debridement. When an obvious callus was visible in the elongation area, usually after 4 to 5 months, the frame was replaced by nail. A total of 16 patients were treated with this protocol, 15 cases were successful, and one case had recurrent osteomyelitis. Replacement of the intramedullary nail or plate may be considered for patients for whom it is inconvenient to carry a frame. However, this protocol may increase the total cost of treatment and the risk of infection. Therefore, further investigation is needed to prove its safety and efficacy.

### Limitations of the study

This study was a single-centre retrospective case series report, rather than a case control study, which provides limited value. In addition, due to the limited number of cases, it was impossible to further analyse and investigate the TFT technique according to the subgroups of age, bone defects size and bone transport type. However, our series contribute successful reconstruction of both massive bone and soft-tissue defects by distraction.

## Conclusion

TFT, in conjunction with soft tissue transport technique, can give good results in most patients (in this article, good and excellent results were observed in 64% of patients). Soft tissue transport is a feasible method in providing good soft tissue coverage on the bone ends. Although it has no advantages over microvascular techniques, it might be an good alternative in the absence of an experienced flap surgeon. Nonetheless, high-quality controlled studies are needed to assess its long-term safety and efficacy.

## Data Availability

The data used and analyzed during the current study are available in anonymized form from the corresponding author on reasonable request.

## Abbreviations

TFT: Trifocal bone transport technique
ASAMI: Association for the study and application of the method of Ilizarov
EFI: External fixation index
AST: Acute shortening and re-lengthening technique
VSD: Vacuum sealing drainage
