# Correction to: Reconstruction of massive tibial bone and soft tissue defects by trifocal bone transport combined with soft tissue distraction: experience from 31 cases

**DOI:** 10.1186/s12891-021-04007-z

**Published:** 2021-02-02

**Authors:** Yong-Qing Xu, Xin-Yu Fan, Xiao-Qing He, Hong-Jie Wen

**Affiliations:** Department of Orthopaedic Surgery, 920th Hospital of Joint Logistics Support Force, Kunming Medical University, 212 Daguan Road, Xi Shan district, Kunming, Yunnan People’s Republic of China 650031

**Correction to: BMC Musculoskelet Disord 22, 34 (2021)**

**https://doi.org/10.1186/s12891-020-03894-y**

After the publication of the article [1] it came to our attention that the wrong versions of Funding was published. Please find below the correct version of Funding.

(Previous version)

**Funding**

The design, data collection and data analysis of the study and interpretation of data and writing of the manuscript were supported by National Natural Science Foundation of China: (H0607).

(Revised version)

**Funding**

The design, data collection and data analysis of the study and interpretation of data and writing of the manuscript were supported by National Natural Science Foundation of China: (81772367).

The original article [1] has been updated.
